# Codesigning health and other public services with vulnerable and disadvantaged populations: Insights from an international collaboration

**DOI:** 10.1111/hex.12864

**Published:** 2019-01-02

**Authors:** Gillian Mulvale, Sandra Moll, Ashleigh Miatello, Glenn Robert, Michael Larkin, Victoria J. Palmer, Alicia Powell, Chelsea Gable, Melissa Girling

**Affiliations:** ^1^ McMaster University Burlington Ontario Canada; ^2^ King's College London London UK; ^3^ Aston University Birmingham UK; ^4^ The University of Melbourne Parkville Victoria Australia; ^5^ Newcastle University Newcastle upon Tyne UK

**Keywords:** codesign, public services, vulnerable populations

## Abstract

**Background:**

Codesign has the potential to transform health and other public services. To avoid unintentionally reinforcing existing inequities, better understanding is needed of how to facilitate involvement of vulnerable populations in acceptable, ethical and effective codesign.

**Objective:**

To explore citizens’ involvement in codesigning public services for vulnerable groups, identify challenges and suggest improvements.

**Design:**

A modified case study approach. Pattern matching was used to compare reported challenges with a priori theoretical propositions.

**Setting and participants:**

A two‐day international symposium involved 28 practitioners, academics and service users from seven countries to reflect on challenges and to codesign improved processes for involving vulnerable populations.

**Intervention studied:**

Eight case studies working with vulnerable and disadvantaged populations in three countries.

**Results:**

We identified five shared challenges to meaningful, sustained participation of vulnerable populations: engagement; power differentials; health concerns; funding; and other economic/social circumstances. In response, a focus on relationships and flexibility is essential. We encourage codesign projects to enact a set of principles or heuristics rather than following pre‐specified steps. We identify a set of principles and tactics, relating to challenges outlined in our case studies, which may help in codesigning public services with vulnerable populations.

**Discussion and conclusions:**

Codesign facilitators must consider how meaningful engagement will be achieved and how power differentials will be managed when working with services for vulnerable populations. The need for flexibility and responsiveness to service user needs may challenge expectations about timelines and outcomes. User‐centred evaluations of codesigned public services are needed.

## 1 INTRODUCTION

Governments around the world face increasing economic and political pressure^1^, ^2^, ^3^ to move towards “coproduction” of public services particularly in health care.^4^ The coproduction concept first appeared in the public administration, civil rights and social care literatures in the United States in the 1970s^5^ and sought to enhance the importance of citizen participation, initially in municipal services such as policing.^2^, ^6^, ^7^, ^8^ The concept rests on an understanding that service users have assets that can help to improve those services^7^ rather than being passive recipients of services designed and delivered by someone else.^1^, ^6^

Although there is no universal definition,^4^ coproduction has been defined as the “… involvement of public service users in the design, management, delivery and/or evaluation of public services”.^7^ A variety of seemingly interrelated terms drawn from different disciplines (eg, cocreation and codesign) have been used which align with principles found in the citizen engagement literature.^1^, ^3^ Advocates suggest that empowering service users and providers to work together can be transformative in creating value in health and other public services, and that service users and communities should play a larger role in shaping decisions and delivery outcomes.^9^ Early work on coproduction sought to acknowledge and enhance the value created by citizens through their engagement with public services.^10^, ^11^

In recent years, coproduction has become a mainstream activity of public sector organizations, particularly in health care and associated social services in many countries.^1^, ^2^, ^3^, ^12^, ^13^ The increasing attention devoted to coproduction and the role of codesign approaches therein is a positive step towards more open and democratic services. However, many public services must strive to meet the needs of vulnerable groups—for example those whose health, economic, cultural or social circumstances produce disadvantage—and whose participation in coproduction or codesign may be restricted. While laudable to seek to collaborate on equal terms with these populations, this is not without challenges.^6^ For example, the mental health literature points to gaps between the rhetoric of service user involvement in international mental health policy and the readiness to adopt such policies in practice.^14^, ^15^, ^16^ Challenges include stigma, poor information exchange and insufficient opportunities for participatory decision making.^14^, ^17^

It is not always clear how coproduction should be carried out in practice with these groups.^6^ Recent work suggests that a lack of critical engagement with issues of power and power relations may lead to circumstances in which coproduction approaches may be harmful.^7^ The literature indicates that, for example, vulnerable groups are under‐represented in patient councils created to give citizens voice in health‐care governance. This may reflect hierarchical structures that require cognitive, communication, conflict management and assertiveness skills that some groups may not have had the opportunity to develop,^18^ or time commitments that are seen as too resource intensive.^2^ The exclusion of vulnerable groups from codesign processes may result in a failure to challenge dominant constructions of health and health care that may unintentionally reinforce oppression and existing inequities. This underscores the need for participatory approaches and supportive institutional contexts in which vulnerable populations can meaningfully engage while developing their individual capacities.^18^

The service design literature,^19^ originating from the participatory design movement in Scandinavia in the 1970s, places priority on designing services for vulnerable consumers.^8^ Codesign arises partly from this literature and partly from the wider coproduction movement.^20^ Codesign recognizes that service users (people with lived experience using particular health, social or public services) are “experts of their experiences”^21^; it aims to use this expertise to improve and develop health and community services based on user needs. Common goals include enhanced user experiences, fewer service design failures and alignment with socially progressive objectives.^8^, ^22^ Codesign draws upon the expertise of service users, but also staff. The process of having these groups working together collaboratively may have additional benefits, in reconfiguring roles and opening up new modes of interaction.^22^, ^23^

Experience‐based codesign (EBCD) is one systematic approach to applying service codesign that was first developed and pioneered as a model to enable improvements in the UK health sector.^24^, ^25^ It has since been adapted to other sectors including education and used in several countries often as part of wider coproduction projects. EBCD combines a user‐centred orientation and a participatory, collaborative and creative change process underpinned by service design thinking.

A two‐day international symposium (the symposium) was held in December 2017 as a research‐funded initiative that brought together 28 practitioners, academics and service users involved in projects to codesign improved services for vulnerable populations in the public sectors of six countries (Australia, Canada, England, India, Scotland and Sweden). Over the 2 days, participants shared case examples of recent service design/codesign applications (many using EBCD) in sectors such as health and social services, employment supports, policing and justice. Our aims were to

- identify challenges when working with vulnerable populations
- codesign improved approaches through roundtable discussions
- formulate a research agenda to advance understanding
- mobilize and share knowledge about engaging vulnerable populations in codesign and coproduction of public services.

This paper outlines the challenges discussed and solutions developed and presents some principles and tactics that codesign facilitators can adopt when working with vulnerable and disadvantaged populations in health and related services.

## 2 METHODS

### 2.1 Case study design

We adopted a modified case study approach^26^ using convenience sampling to elicit and analyse the challenges of codesigning with vulnerable populations. The research team drew upon their knowledge of the research literature and projects that applied codesign and coproduction approaches to improve public services with vulnerable and disadvantaged population in their jurisdictions (Canada, England, Australia), as well as other countries (New Zealand, Scotland, Switzerland, United States).

Each case is based on practitioners’ experiences of codesign in public services. During the event, three panels of project leads presented a total of 11 illustrative cases of working with vulnerable populations. We present quotes from symposium participants using a code [case number (as listed in the first column of Table 1), followed by source—video (V) or template (T)] to preserve confidentiality. Each panellist shared high and low touchpoints (positive or negative experiences) from their own case. For this paper, we selected eight cases that met the following criteria:

**Table 1:** Overview of cases

Population	Public service	Project aim	Country	Time frame
(1) Adults with mental health problems	Community Health and Social Services	To test the effectiveness of Mental Health Experience Codesign in improving recovery for service users, quality of life for carers and attitudes towards recovery of staff	Australia	June 2013—August 2017
(2) Adults with mental health problems	Community Health and Social Services	Making recovery real initiative. The goal is to ensure that people who have experienced the challenge of mental health conditions are listened to, and that their experiences are valued. In drawing upon the lived experiences of people with mental health issues, services and support can be developed to help people to take control of their recovery, and to enjoy full, satisfying lives.	Scotland	November 2015—on‐going
(3) Adults with personality disorders	Ambulance Services	To identify crisis responses that help or hinder persons with borderline personality disorder, ambulance crews and call centre staff, and to design feasible solutions to improve experience and relieve pressures on staff	England	March 2015—on‐going
(4) Youth with mental disorders	Health, housing, CAS, Case Coordination, Community Mental Health	To codesign improved experiences of youth mental health service coordination and transitions to adult services	Canada	March 2016—September 2017
(5) Young workers with mental health issues	Employment Support Services, Community Services for Youth	To codesign improved employment supports to make it easier for young workers with mental health issues to find and maintain employment	Canada	January—December 2017
(6) Survivors of domestic violence	Police Services	To understand and design improvements to address dissatisfaction with police response to domestic violence by working with police and survivor representatives	England	October 2016—February 2018
(7) Young offenders	Justice Services	To understand the experiences of young people with mental health problems in the youth justice programme and codesign justice and social services improvements to deliver needed supports to youth	England	November 2016—April 2018
(8) Indigenous populations	Indigenous Health Policies	Through community‐based participatory research (CBPR), to analyse the shift and support design of Indigenous health policies in Canada from government defined towards community controlled	Canada	March 2009—May 2014

- Service users and service providers working together to codesign or coproduce a health or other public service;
- Service users were members of a vulnerable and disadvantaged population(s);
- Methods and approach consistent with the active, on‐going involvement of participants in a non‐hierarchical way in codesign or coproduction; and
- Directly related to service design.

Table 1 provides an overview of the selected cases with respect to population, service and jurisdiction. The various vulnerable and disadvantaged groups included young workers, youth and adults with mental disorders or personality disorders and their carers, survivors of domestic violence, and Indigenous peoples. Public services included health care, community mental health, police, justice and employment support services. The cases varied in scope from local initiatives to a full‐scale cluster randomized controlled trial (CRCT) in the case of adults with mental disorders in Australia.^27^

### 2.2 Event participants

The symposium participants included six service users from vulnerable and disadvantaged groups, six service providers, 11 researchers/project leads for the presented cases and other academic participants with experience in service codesign/coproduction with vulnerable populations from other countries (eg, Switzerland and Sweden). Collectively, researchers represented multiple disciplinary backgrounds (Health Policy, Occupational Therapy, Applied Psychology, Health, Aging and Society, Business, Design, Applied Ethics, Epidemiology and Organizational Sociology).

### 2.3 Theoretical propositions

Prior to the event and drawing on the existing published literature, we hypothesized that a number of distinct challenges would emerge for vulnerable and disadvantaged populations in different contexts that would require special attention. We expected that

- Identification, recruitment and on‐going engagement of participants from vulnerable groups would be challenging because of the nature of their condition/circumstances^2^, ^18^;
- Accommodations for health or other conditions would influence engagement activities^28^;
- Economic considerations would be required to enable participants to engage^18^, ^28^;
- Power differentials would require particular attention in the codesign process^18^, ^28^; and
- Funding challenges would arise because of low visibility and relative lack of advocacy organizations.^18^

### 2.4 Data sources

There were two main data sources collected over two phases. First, in advance of the event, we created and emailed a data collection template to project leads for each case. Seven templates were completed and returned to the lead author. The template asked about project rationale, vulnerable group, coproduction/codesign approaches adopted, key touchpoints (emotional highs and lows in the project), challenges and lessons learned about engaging with this group, broader public engagement strategies and suggestions for future research. The templates were used to create summaries that were pre‐circulated to participants as preparation for the symposium.

The second data source was a record of content presented and discussed during the symposium about the various cases. Following each panel, participants were divided into small groups to engage in a collaborative codesign process for one of the selected cases. Facilitated small group discussions began by deciding on a particular problem that needed to be addressed based on the case presentation. Participants then individually and collectively brainstormed potential solutions. The facilitator and group members recorded discussion content. Each group arrived at a problem statement, a visual prototype and written description of their solution, which were shared with the whole group. All notes taken at the symposium were transcribed to electronic format. The presentations of the problems, solutions and prototypes to the large group and all large group discussions were summarized on flipcharts and video recorded. Videotaped content was transcribed verbatim by two of the authors (GM and AM) and integrated with electronic notes of the flipchart content.

### 2.5 Analysis

Following the event, small and large group discussion and template data were synthesized to create individual case summaries that included the problem statement, the proposed solution, key discussion themes, a visual prototype and description. All case summary content was reviewed following a thematic analysis approach to identify common and shared themes which pertained to (a) challenges of codesigning with vulnerable and disadvantaged populations; (b) principles of codesign when working with these groups; and (c) tactics to achieve these principles. In the cross‐case analysis of challenges, the lead author used pattern matching to search for confirming and disconfirming evidence for the pre‐specified theoretical propositions.^26^ This involved three authors (GM, AM and SM) comparing themes from the discussions at the symposium for each case with the prior theoretical propositions drawn from the literature and identifying statements that supported or contradicted the propositions. We then tabulated the cases that supported or contradicted each proposition. Next, the same three authors independently identified principles and tactics raised during the codesign activities for each case using an inductive approach and met to discuss these until consensus was reached. The lead author then created a summary of the overarching principles and tactics. The case summaries and all identified themes were member‐checked and revised based on symposium participant feedback.

Boxes 1, 2, 3 present three illustrative case examples that offer diversity with respect to (a) codesign stage (recruitment, on‐going engagement and implementation); (b) population served (young offenders, adults with mental disorder and youth with mental disorder); and (c) geographic location (United Kingdom, Australia and Canada). The examples are based on (a) challenges in study recruitment for youth justice services in the United Kingdom; (b) implementation of community mental health service improvements in Australia; and (c) on‐going engagement in youth mental health service coordination in Canada.

Box 1 **Direct engagement and support for youth participants**

1 *The Challenge:* In the case of justice services for young people who offend in England, the overarching difficulty discussed was gaining access via gatekeepers to the youth population in order to help them engage in EBCD. Current legal and ethical frameworks applied to this vulnerable population group can also prohibit their participation in research. *Key Discussion Points:* Participants discussed how organizational barriers such as service providers’ own ability and capacity to “open the door” are being affected nationally by government policies aimed at downsizing and devolving youth justice services, and at a more local level feeling of being “over‐researched” and mistrusting the research process itself. Participants suggested the need to adopt alternative recruitment strategies such as engaging with third‐sector organizations and groups that may work with young people who are at risk of or on the cusp of offending. Participants also felt that it was important to meet youth where they are—spatially in informal community settings and digitally through the online community. Creating a youth panel specific to the project and incorporating support mechanisms were additional suggested approaches to encourage participation and support the research process. *The prototype:* An approach that directly engages with youth rather than recruiting through justice services. This could include engagement with third parties and youth‐led groups in the community to participate and working with family members to provide a support system for youth engagement. This joined‐up approach could help better navigate legal and ethical frameworks and increase participation.

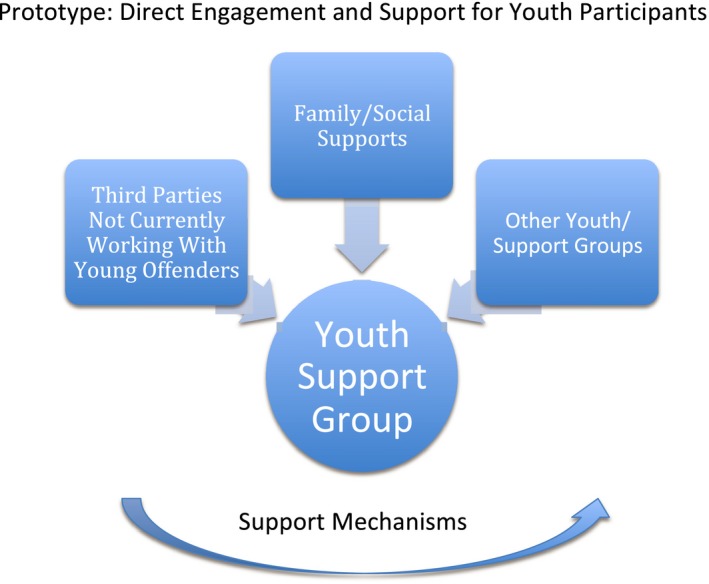

Box 2 **Getting beyond experience and tackling implementation challenges**

1 *The Challenge:* The panellist presented this challenge based on results from testing mental health experience based codesign as a complex intervention within a cluster randomized controlled trial in non‐clinical recovery services for adults experiencing severe mental illness. The panellist discussed the challenge of connecting EBCD approaches more closely with implementation science following codesign stages.

*Key Discussion Points:* Event participants noted the importance of establishing parameters around what is changeable and connecting this with what')s doable. Training (or orientation) in the codesign process was seen to be an essential preparatory step. Participants recommended enhanced connection by developing implementation plans that prototype responsibilities and set out targets. Further inclusion of methods such as the critical incident technique (identifying features for success) or role‐plays in codesign might foster more implementation capacities. A role‐play example might involve codesign participants using play money to decide on resource allocation across the different parts of the implementation process. There was a strong sense that building service user, carer and staff capacities is important to foster greater potential for service users and carers to remain involved within the implementation stages that follow on from codesign. Keeping track of these processes and sharing positive and negative impacts of implementation is needed.

*The Prototype:* An embedded model of continuous learning would include attention to what has worked in other studies and activities for implementation capacity building. Such a solution would include development of implementation plans within codesign.

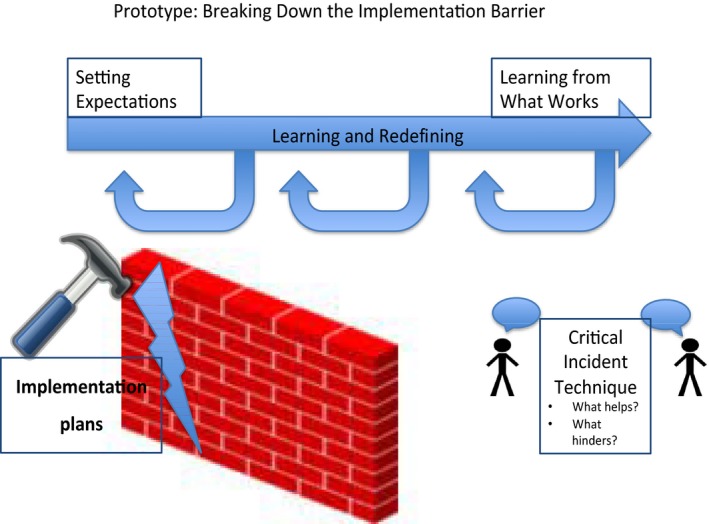

Box 3 **Addressing on‐going engagement challenges**

1 *The Challenge:* In the case of coordination of youth mental health services in Canada, the panellist raised the challenge of keeping youth engaged on an on‐going basis through the various phases of the codesign process. While it was difficult to keep youth engaged in the work, those who remained engaged found the process extremely valuable. *Key Discussion Points:* Event participants talked about the challenge of articulating the “magic” of codesign, and wanting to understand the “secret sauce” that makes it work, in order to motivate continued engagement. Symposium participants recommended a continuous evaluation process, with opportunities to check‐in with study participants in a fluid and individualized way throughout the codesign process. Youth could state their goals and help to develop evaluation measures at baseline and continue to choose among anonymous or face‐to‐face modalities through which to provide their feedback that are flexible, fluid and natural. Tactics may include story‐telling, providing informal comments on sticky notes or online following each event or creating a reflective video as a group. Another recommendation was to involve peers in evaluation activities to build relationships that encourage honest and deep reflection. There was a strong sense that traditional quantitative measures will not be appropriate. There is a need to consider motivators and recognition for youth participation that go beyond basic honoraria, and also to recognize that traditional ethics forms may be intimidating for vulnerable populations.

*The Prototype:* A youth‐driven approach to on‐going evaluation to help articulate the value of participation in codesign as a basis for encouraging on‐going youth engagement.

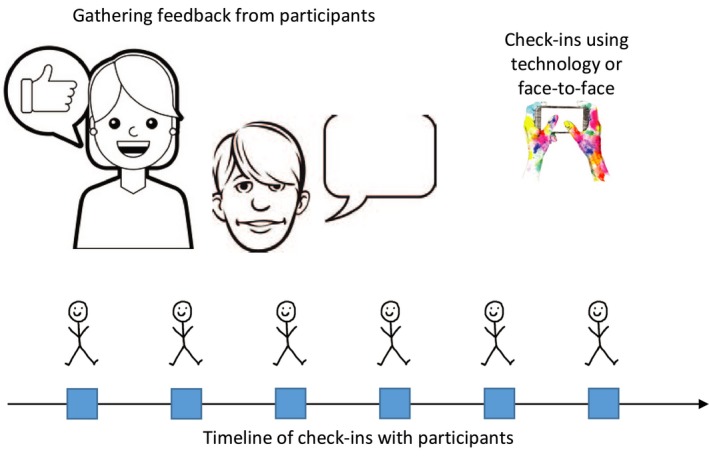

## 3 FINDINGS

### 3.1 Challenges, principles and tactics when codesigning public services with vulnerable populations

Table 2 presents the key challenges identified for each case and compares them to the theoretical propositions. Three stood out as common across almost all cases: issues with initial recruitment, repeated engagement and power differentials. Health considerations were notable in the cases involving youth and adults with mental health issues and personality disorders, and funding challenges were noted in five cases. Economic and social considerations were mentioned in all three cases involving youth (youth employment, justice and mental health services).

**Table 2:** Pattern matching to a priori propositions: challenges working with vulnerable and disadvantaged populations

	Recruitment	Repeated engagement	Health concerns	Economic and social circumstances	Power differentials	Funding challenges
Adult Mental Health Services						
(1) Australia	✓	✓			✓	✓
(2) Scotland	✓	✓			✓	✓
(3) Ambulance Services			✓		✓	
(4) Youth Mental Health Service Coordination	✓	✓	✓	✓	✓	✓
(5) Employment Services for Young Workers	✓	✓	✓	✓	✓	✓
(6) Police Services for Domestic Violence	✓	✓			✓	✓
(7) Youth Justice Services	✓	✓		✓	✓	
(8) Indigenous Populations	✓	✓			✓	

#### 3.1.1 Engagement

All cases had challenges establishing initial and sustaining repeated engagement over the course of the projects. Symptoms and life circumstances interfered with some people's ability for prolonged engagement. Projects involving youth with mental health issues noted high dropouts due to health crises, housing transitions and service closures.

… youth were going into crisis and having to leave the area for treatment. Some youth had to transfer to a different foster home, community, or move to a different province because they'd aged out of youth housing. Some were kicked out of a program [for] not complying with the rules. [4V]

In the Australian study of adults with mental illness, the greatest challenge was identifying carers (ie, friends and family in a caring relationship to the person) for some adults with mental illness. Only half of the study participants reported having carers, and services were not able to provide accurate, up to date contact information for them, suggesting “a need for specific engagement efforts for carers.” [1T]

In the study involving domestic violence survivors, local participants were less willing to come forward and participate because “they were really scared and worried” [6V]. In the young offender study, recruitment proved impossible due to legal anonymity for young offenders prior to age 18, and a reluctance of staff members to act as gatekeepers in the research, “…straight away I got ‘we don't have capacity for this; we're too busy.’” [7V]

Box 1 is a summary of the challenge, the discussion and the solution prototype as it emerged at the symposium in the case of youth justice services in England.

#### 3.1.2 Power differentials

Power differentials were challenging in all of the cases. For example, when working with adults with mental disorders, the Australian study noted that “challenges occurred in the dynamics between service users who had had negative experiences, and staff within working groups” [1T]. Similarly in Scotland, tensions were noted due to dominance of the medical model vs a recovery model that places lived experience of service users at the centre because “… we are questioning that dominant medical world and they aren't liking it—they are struggling with it.” [2V]

In England, adults with personality disorders (Case 3) felt highly vulnerable when reflecting on prior experiences of apparent powerlessness. Power issues also had to be negotiated between youth with mental health issues and their former service providers prior to the codesign session (Case 4). Tensions also arose for domestic violence survivors when police services would only permit participation of women within the region of the study (Case 6). Finally, in studies involving Indigenous communities, historical legacies of discrimination and harm, and “a deep on‐going history of colonialism that still persists…within our health care system…,” can influence the acceptance of non‐indigenous researchers and make research difficult in these communities [8V].

#### 3.1.3 Health concerns

In several cases (3, 4, 5, 6), health vulnerabilities affected participation in codesign processes. For example, in the project involving work with domestic abuse survivors, recounting experiences sometimes triggered past trauma. In some studies, unexpected personal issues (eg, illness “flare‐ups” or medication issues) interfered with participation in focus groups or codesign meetings. In one case, “a youth participant at a codesign event had discontinued all her medication two days before (cold turkey) and was ill at the event.” [4T] Other health concerns (eg, social anxiety disorder or medication side effects) limited participants’ abilities to fully contribute to codesign discussions, or feel comfortable at events. For example, one researcher explained,

How and where we offered food and refreshments … we didn't realize it would be problematic, but some youth had eating disorders and felt very uncomfortable eating in front of other people. [4V]

#### 3.1.4 Economic and social circumstances

Economic and other social challenges such as difficult home circumstances, being precariously housed or precariously employed, prevented consistent participation for the studies involving youth (cases 4, 5, 7).

Youth with mental health and employment challenges face many barriers to engaging in the healthcare system and sustaining employment. They are focused more on the ‘day‐to‐day’ concerns of life, and may not see immediate value in participating in ‘codesign’ or projects focused on system‐level change. [5T]

#### 3.1.5 Funding Challenges

Working with vulnerable and disadvantaged populations was also highly resource intensive. It took considerable resources and a dedicated research coordinator to reach out, provide information, support travel and build relationships with adults with mental health issues in Australia (case 1) and youth with mental disorders in Canada (case 4). In the young workers’ project, “Building capacity of the youth to participate takes time, patience and nurturing” [5T] to prepare them to participate in the research. In the project for survivors of domestic violence (case 6), significant concerns arose about the ability to allocate sufficient resources to the external team. Securing external resources to bring in designers in order to “dream big” [1T] about possible service improvements and to provide resources for further development and to support implementation was also a challenge in the study of services for adults with mental health issues in Australia (case 1).

#### 3.1.6 Other

Additional challenges in carrying out codesign work with specific populations included ethical considerations, context and communication. Some study participants shared stories of traumatic experiences that were very upsetting for research team members to hear (case 4). Well‐intentioned research ethics processes inadvertently created anxiety for some vulnerable populations; for example, concerns about trust and exploitation were particularly acute for Indigenous populations (case 8). In mental health contexts, poor communication between services, service users and carers presented challenges in two studies (Cases 1 and 4).

Concerns were also expressed that more attention needs to be given to how to support implementation and evaluation of the changes resulting from codesign processes through using approaches such as the critical incident technique,^29^ so that vulnerable populations who participated in codesign with the hope of making tangible improvements to services are not disappointed. Continuing to involve the people who codesigned the improvements is another challenge during implementation (see Box 2).

### 3.2 Lessons learned

Table 3 presents the results of the cross‐case analysis as it pertains to suggested solutions or strategies to address these challenges. A frequent recommendation was the need for flexibility by following a set of principles or heuristics rather than pre‐specified steps. The table presents the common principles that emerged across cases in bold type and relevant cases in brackets. Bullet points list tactics suggested by event participants that align with specific challenges, but the principles may also apply more broadly. This combination of principles and suggested tactics may assist practitioners working with vulnerable populations in other public service design projects.

**Table 3:** Lessons learned across cases

Challenge	Principles
Recruitment	Build on Trust (1, 5, 6, 7, 8) Engage an “insider” as a champion (6) Recruit through established networks, informal groups, voluntary or “outside the box” organizations, use peer to peer approaches, targeted social media (1, 2, 3, 4) (5, 6, 7, 8) Engage with participants in advance of research processes (1) Flexibility and Responsiveness (4, 5, 6, 7, 8) Have flexible participation options (in‐person, Skype, email, online) using a variety of media for data (art‐based, music, crafts, visual diaries, photographs) (5, 6, 7, 8) Bring codesign process to informal community spaces or online (5, 8)
Repeated engagement	Mutual Understanding (1, 3, 4, 5, 6, 8) Foster solid relationships among research team, decision makers and participants (1, 4, 5) Understand different motivations, examples of what is possible and acknowledge needs that cannot be met (4, 5, 6, 8) Agree to a shared vision as a central purpose that guides the project (8) User Centredness (4, 5, 3, 6, 8) Focus on community/user‐identified needs (not researcher or system identified) (8) Fully understand lived experience through conversation (6) Prioritize people over process (objectives or timelines) (3) Reciprocity (3, 4, 5, 6, 7) Assess individual skills and capacity to participate, offer training and support that help build capacity (4, 6, 7) Have a stable group to offer support that people feel part of (3, 5, 7) Ensure meaning and purpose for participants and that process is making a difference (3, 7)
Power differentials	Empowerment (2, 3, 5, 6, 8, 7) Specify shared roles and responsibilities to empower community members (6, 8) Encourage participants to recognize that they are making a difference (2, 5) Constantly take stock of user perspective so staff do not take over, listen to voices of people with lived experience first who drive the process (3, 6) Consider that unpaid volunteers may feel greater freedom to voice opinions (3) Adopt non‐stigmatizing options for data sharing (4, 5) Power Sharing (3, 8) Formalize agreements for shared ownership of data and protection of Indigenous knowledge (8) Communicate openly with respect to documents, data and reporting (8) Share leadership with a willingness to be challenged and directed (6, 8) Establish an expert panel to address stalemates and provide advice (6)
Health concerns	Trust in Process (1, 3, 4, 5, 6, 8) Recognize and foster trust as participants relive trauma (3) Recognize staff vulnerability and fear of meeting the “other” Offer joint training to build mutual understanding (3) Conducive Environment (4, 5) Codesign a “Comfort Agreement” for rules of engagement (4, 5) Create space for people to share their angst before moving to codesigning improvements (3, 4, 5) Provide emotional support and a quiet space for retreat at meetings, have a professional present where appropriate (4, 5, 6) Recognize Emotional Toll (3, 4, 5) Over‐recruit most vulnerable participants (4, 5) Address safety needs of team and participants by offering debriefs, building in time and resources, and waiting for participants to be ready to share (3, 4, 5) Take time to build organizational readiness to hear feedback (3)
Economic and social circumstances	Understand and Respect Culture Differences (3, 6, 8) Take time to bring everyone together (10) to address tensions in worldviews (eg, statistics vs lived experience); (6) acknowledge and honour different ways of knowing (2) Use knowledge sharing approaches that are comfortable (eg, sharing circle in Indigenous communities) (8) Establish cultural safety (8) Understand the Person in their Context (3, 4, 5, 6, 8) Take time to learn about history and context of the various groups involved (6, 8) Facilitate transportation by sending taxis to pick up most difficult to engage groups and provide videoconferencing or online options (4, 5) Vary meeting times (morning, evening, lunch, weekend) to maximize participation across several meetings; offer flexibility in attendance (4, 5, 6) Have peers check in on peers, use user friendly language (3, 4, 5, 6)
Funding challenges	Build Credibility (7) Consider lived experience as legitimate evidence of health system impact (7) Secure champions from the medical community to advocate with funders for uptake (7) Partner with social health researchers (7) Demonstrate Impact (7) Build a case to garner support from funders/system administrators (7) Diffuse cocreated evaluation tools throughout systems to increase uptake (7) Be Opportunistic (3) Be ready to engage in coproduction when opportunities arise (partners, recourses, readiness) (3) Be flexible and responsive to funding challenges (3)

**Figure 1:**
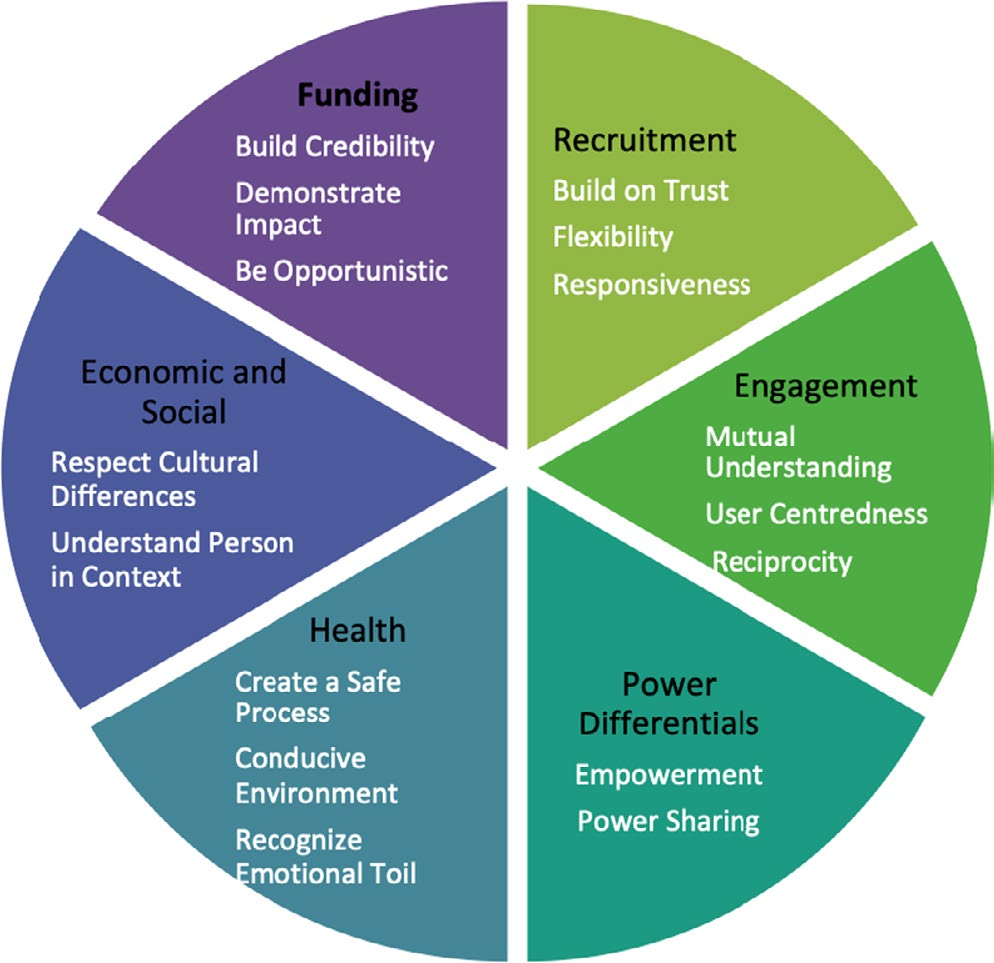
Challenges and principles for codesigning health and social services with vulnerable populations

We summarize the challenges and principles in Figure 1:

#### 3.2.1 Engagement

Trust, flexibility and responsiveness were identified as important principles in the recruitment processes. Participants recommended that recruitment be built on pre‐existing trusting relationships (eg, lawyers for victims of domestic violence, peer groups for youth living with a mental illness). In Indigenous communities, it is essential to identify and work with a gatekeeper and take time to understand issues as defined by the community rather than taking an outside perspective.

Other key tactics included being responsive to what works for the group, adopting creative ways to reach out, such as targeted social media, engaging “insider” champions, leveraging established networks and engaging natural community‐based groups. In the Young Worker's study, “It was the ‘outside of the box’ … innovative employment providers that were willing to engage in a dialogue about system change” and “appreciated the opportunity to reflect and engage in dialogue.” [5T]

Continued engagement requires time to foster mutual understanding, consider unique contexts and motivations, give primacy to the user or group experience, ensure meaning and build capacity (see Box 3). It is essential to take time to fully understand experiences, letting go of timelines should they interfere with relationships or group functioning. For example, in ambulance services, it was important to “Understand the heavy pressure on crews, and give them time to elaborate [on this] before going forward.” [3T]

#### 3.2.2 Power differentials

Empowerment of service users and power sharing across perspectives was noted as essential and very rewarding,”… what was really amazing was when youth presented their prototypes—family members and service providers were just blown away by what youth had presented”. [4V] specifying shared roles and responsibilities and listening to service user voices throughout codesign processes resulted in “…relationships developing and conversations changing between family members and service providers that had sometimes been adversarial.” [4V]

Formal agreements are often advisable, but an Indigenous research protocol such as OCAP^®^ 30 that upholds community ownership, control, access and possession of research knowledge generated within the community is required when working with Indigenous populations. This can protect against historically harmful research approaches and encourage inclusion of researchers within the community. As in other groups, leadership must also be shared in Indigenous communities, and leaders must be willing to be challenged and directed by community members, rather by pre‐conceived notions about issues and approaches.

As a non‐indigenous person‐ I negotiate my position, my privilege and my power in performing research with indigenous peoples. This … begins with being open to working with and acknowledging the problems and needs of the community first. [8V]

To avoid unresolvable differences between those at different levels of authority (eg, police and survivors in the domestic violence study), a recommended tactic was to establish a separate advisory panel representing all perspectives to provide advice and recommendations.

#### 3.2.3 Health concerns

Creating processes that (a) enable participants to feel safe, (b) establish an environment conducive for codesigning services and (c) recognize the emotional toll that codesigning services can have on participants and researchers were important principles to address health concerns. Principles of engagement can be designed to facilitate open and safe conversation and allow space for venting early on in the process so that later codesign stages can be more productive; this was a specific element in the training delivered in the Australian codesign study. There is also a need to be responsive to participant needs, offer frequent breaks and provide a quiet space (or “chill room”), and emotional support should participants become distressed. Working within existing groups is a suggested tactic to offer natural support.

It is also important to recognize that distress may also be an issue for staff members who may feel quite anxious about meeting clients who are in a position to be critical of their practices.

A lot of health staff seem far more interested in their status and control than in people's lived experience in the end…. people who make decisions … they are particularly uncomfortable about valuing life experience. [2V]

Practitioners need to support staff as well as service users and keep the emphasis on improvement rather than past difficulties. Then, staff have the opportunity to discuss the challenges they are facing. In the ambulance service case, “Staff has really needed to talk about their touchpoints so it was incredibly useful…” [3V].

#### 3.2.4 Economic and Social Circumstances

Understanding the history and context of each group and respecting cultural differences enable knowledge to be shared in a culturally safe manner. Specific tactics such as sending taxis to pick up participants and providing videoconferencing or online options for groups whose circumstances make it difficult to engage (eg, youth with mental health issues) may facilitate participation.

Youth have varying degrees of connection with formal systems and challenging life circumstances as well as episodic mental health issues [that] make it difficult to establish a consistent and meaningful connection. [5T]

Similarly, varying meeting times (morning, evening, lunch and weekends) and offering shorter meetings can maximize participation across several events rather than asking participants to attend every meeting.

#### 3.2.5 Funding challenges

To garner support from funders for more widespread use of coproduction in service design, participants emphasized the importance of evaluation to demonstrate impact. They also suggested securing champions from the medical community and other professional groups. Since funding support for public services for vulnerable groups is often in flux, practitioners and researchers were advised to be flexible and remain vigilant to embrace opportunities for funding, resources and partnerships that may arise.

Because the teams were being disbanded, funding taken away– one person was in a phone call saying “I've got a case load of 12 young people that I have no one to give them to”. And there is me [researcher] going, “Oh can you just find me five young people?” So I decided to withdraw then and say the door is always open … but I am not going to push any further. [7V]

## 4 DISCUSSION

The eight case studies illustrate a number of common challenges that were consistent with our theoretical propositions when codesign health and other public services with vulnerable groups and themes that have emerged in prior literature.^31^, ^32^, ^33^, ^34^, ^35^, ^36^ Engagement challenges^9^, ^12^, ^20^ and power differentials were acutely and consistently felt when working with these populations.^18^, ^37^, ^38^ In most cases, study participants were experiencing some combination of challenging health, social and financial circumstances, or stigma associated with their social identities (eg, lived experience of mental illness, young offender, domestic violence survivor or being member of an Indigenous population). Practitioners should consider intersectionality of vulnerabilities^28^ since discrimination from multiple sources can combine to unintentionally perpetuate service user marginalization when designing health and social services.^39^

A cross‐cutting theme was the centrality of relationships to the entire codesign process^27^ and the need to be flexible and responsive to participants’ needs. Event participants consistently recommended following a set of core principles, rather than a series of rigid steps; taking time to fully engage, listen for understanding and not move forward until participants or communities are ready. Otherwise the trust and meaning so necessary to the process will be lost. It also means finding formal and informal ways to ensure power is shared, the voices of vulnerable groups are given precedence in planning, design and evaluation, and that processes are reflective of a deep understanding of the user context. Given the vulnerability of participants, special attention should be paid to accountability and transparency. There may be a greater need than usual for formalizing rules and expectations for the design work and being explicit about responsibilities for implementation. Extra attention to transparency during recruitment can ensure participants clearly understand what is expected, and on‐going communication from practitioners can promote trust and model the open attitude and willingness to learn that is needed for effective codesign.^24^

The reflections from practitioners of codesign processes with different vulnerable populations offer a set of principles and suggested tactics that others can adopt for service design with vulnerable populations. Practitioners need to be vigilant in protecting vulnerabilities, while simultaneously empowering participants to codesign improvements based on challenging past experiences. Attending to the “human side” can be difficult yet simultaneously the most rewarding part of codesign practice with vulnerable populations. Practitioners must navigate the need for formal accountability while retaining flexible and responsive processes. New understanding is required of “downward” accountability that acknowledges “partnerships and complexity” rather than the “upward accountability” to predetermined organization goals and outcomes that is the traditional logic of many health services.^39^

From an institutional perspective, the findings also suggest that the procedures of health‐care organizations and professional regulators can present challenges to researchers doing codesign work. It is essential that good ethics practices protect those sharing experiences, without overwhelming them. Research ethics boards must be supportive of Indigenous research methods and frameworks, and ground rules should be established to ensure cultural safety.^40^

Symposium participants expressed appreciation for the opportunity to come together to share challenges and successes to date and to plan for future opportunities to continue to learn together how to improve codesign practice with vulnerable populations. A high priority for future deliberations is how to support implementation of codesigned solutions in service delivery, as an integral part of the design efforts^23^; to honour the contributions participants have made by sharing often difficult experiences. Setting realistic expectations and adopting a stance of continuous learning can help to break down implementation barriers. Meaningfully involving service users in choosing outcomes for evaluation and in codesigning evaluation tools and approaches were considered high priorities for future research.^22^, ^27^

Important considerations for integrating these approaches into the mainstream way of doing business in health services were resource intensity and planning for the unanticipated. All researchers described this process as resource intensive, requiring intensive efforts by one or more research coordinators for ethics (sometimes at more than one organization), recruitment, continued engagement, data gathering, data analysis and codesign meetings. When considering how to embed coproduction in day‐to‐day health service design, budgets must cover time of designers and offer fair honoraria for participants’ time, with room for unanticipated contingencies.

### 4.1 Strengths and limitations

A strength of this work is that the event format allowed for learning from multiple cases of working with vulnerable populations to codesign public services. A limitation is that each case could be further explored in much greater depth to obtain a more fulsome understanding of what worked well and what could be improved. This will be the strategy in future meetings of the event participants. The on‐going international collaborative will play a continuing role in developing practitioner‐led applications of health and social service design and improvement approaches.

## 5 CONCLUSION

Lessons from the eight cases examined at our international symposium suggest that challenges in engagement and power differentials require particular attention when codesigning health and other public services for vulnerable populations. Our analysis prioritizes a set of principles that can enhance engagement and create flexible and responsive codesign processes that are respectful of the readiness of vulnerable populations. In future, greater emphasis is needed to support implementation and user‐centred evaluation of codesigned services to demonstrate effectiveness. Suggested approaches for vulnerable populations can help to overcome stigma, create safe spaces and support participants who might have experienced trauma, while respecting principles of control and access to the knowledge gathered from these communities. On‐going work of the international consortium and future research will better connect civil society in health and social service design, while improving transparency and accessibility of services for all citizens, including those most vulnerable.

## CONFLICTS OF INTERESTS

The authors have no conflict of interest to declare.

## ETHICS APPROVAL

Each case study had received the necessary ethical approvals where required in their respective countries. All symposium participants completed a video release form giving permission to use their video commentary for analysis and knowledge exchange purposes. The McMaster University Research Ethics Board did not require ethics approval for the symposium, and no original data collected in the individual studies were shared.
